# Cardiac fibromas in adult patients: a case series focusing on rhythmology and radiographic features

**DOI:** 10.1093/ehjcr/ytae410

**Published:** 2024-08-08

**Authors:** Karl Finke, Thorsten Gietzen, Daniel Steven, Stephan Baldus, Henrik ten Freyhaus, David Maintz, Lenhard Pennig, Carsten Gietzen

**Affiliations:** Department III of Internal Medicine, Heart Center, Faculty of Medicine and University Hospital Cologne, University of Cologne, Kerpener Street 62, 50937 Cologne, Germany; Department III of Internal Medicine, Heart Center, Faculty of Medicine and University Hospital Cologne, University of Cologne, Kerpener Street 62, 50937 Cologne, Germany; Department III of Internal Medicine, Heart Center, Faculty of Medicine and University Hospital Cologne, University of Cologne, Kerpener Street 62, 50937 Cologne, Germany; Department III of Internal Medicine, Heart Center, Faculty of Medicine and University Hospital Cologne, University of Cologne, Kerpener Street 62, 50937 Cologne, Germany; Department III of Internal Medicine, Heart Center, Faculty of Medicine and University Hospital Cologne, University of Cologne, Kerpener Street 62, 50937 Cologne, Germany; Institute for Diagnostic and Interventional Radiology, Faculty of Medicine and University Hospital Cologne, University of Cologne, Cologne, Germany; Institute for Diagnostic and Interventional Radiology, Faculty of Medicine and University Hospital Cologne, University of Cologne, Cologne, Germany; Institute for Diagnostic and Interventional Radiology, Faculty of Medicine and University Hospital Cologne, University of Cologne, Cologne, Germany

**Keywords:** Cardiac fibroma, Cardiac masses, Premature ventricular contractions, Case series, Magnetic resonance imaging

## Abstract

**Background:**

Fibromas are rare primary benign cardiac tumours that can become symptomatic due to expansive growth, ventricular rhythm disturbances, and sudden cardiac death. Distinguishing fibromas from other (malign) cardiac masses is essential for accurate diagnosis and treatment. While there is some experience in management of cardiac fibromas in children, management of adult patients is unknown.

**Case summary:**

We present three cases of cardiac fibroma in adult patients diagnosed by echocardiography, cardiovascular magnetic resonance (CMR), and computed tomography (CT): (1) a 55-year-old male with a left ventricular fibroma leading to reduced left ventricular ejection fraction and mitral regurgitation. He had family history of sudden cardiac death, showed premature ventricular contractions (PVCs), and was treated with a primary preventive subcutaneous implantable cardiac defibrillator (S-ICD); (2) a 39-year-old male with right ventricular fibroma as an incidental finding. He complained of episodes of PVC. Due to a low PVC burden, decision was made against ablation and the patient was planned for follow-up; and (3) an 18-year-old female with left ventricular apex fibroma detected by CMR shortly after birth and confirmed by surgical biopsy. Being asymptomatic, conservative management was pursued and follow-up by CMR planned.

**Discussion:**

Cardiac fibromas can show various clinical presentations and hence being detected late in life. Given potential complications of surgical biopsy, diagnosis of cardiac fibromas is primarily based on echocardiography, CT, and CMR. Rhythm disturbances as PVCs are common. Due to association with ventricular arrhythmias and sudden cardiac death, preventive ICD placement might be appropriate on an individual basis.

**Learning points**

- Cardiac fibromas may present with various clinical phenotypes and rhythmology.
- Diagnosis of cardiac fibromas primarily relies on echocardiography, cardiovascular magnetic resonance (CMR), and computed tomography as non-invasive imaging methods with CMR being the gold standard to distinguish fibromas from other cardiac tumours.
- Preventive implantable cardiac defibrillator placement might be warranted on an individual basis considering size, impairment of cardiac function, risk of ventricular arrhythmias, and sudden cardiac death if cardiac fibromas are not feasible for surgical resection in adult patients.

## Introduction

Primary cardiac tumours are rare, with described frequencies of 0.02% in autopsy series.^1^ Cardiac fibromas are the second most common benign primary cardiac tumours in children, with an extremely rare occurrence in adults.^2^ While consisting mainly of fibroblasts and collagen, margins of cardiac fibromas infiltrate the surrounding myocardium.^3^ Fibromas are mostly found in the left ventricular free wall, followed by the right ventricle and the interventricular septum.^4^ Although benign, fibromas can lead to cardiac symptoms due to expansive growth causing left- or right ventricular outflow tract obstruction, impairment of valve function, and disturbance of the cardiac electric conduction system.^5^ Furthermore, cardiac fibromas are associated with ventricular arrhythmias and sudden cardiac death, most likely due to infiltration in the surrounding myocardium interdigitating with cardiomyocytes.^4,6^

Patients might be clinically asymptomatic with fibromas as an incidental finding or symptomatic with dyspnoea, chest pain, palpitations, or syncope induced by tachyarrhythmias or as cardiac arrest.^4–6^ It is estimated that ∼10% of cardiac fibromas are detected post-mortem upon autopsy after sudden cardiac death.^7^ Compared to rhabdomyomas, cardiac fibromas do not regress spontaneously.^5^ Differential diagnosis for cardiac fibromas includes rhabdomyomas in children and myxomas, thrombus, or lipomas in adults.^8^

Once diagnosed, there is no evidence-based or guideline-directed approach for management of cardiac fibromas. However, in children and adolescents, a fibroma should be excised even if asymptomatic to prevent malignant arrhythmias, if operable.^5,6^ The diagnostic and treatment approach to fibromas in adult patients is less defined, and there are only case studies describing cardiac fibromas in adult patients.^9,10^

We present three adult patients with cardiac fibroma diagnosed by multimodality imaging with one case being confirmed by biopsy and their subsequent clinical management.

## Summary figure

**Figure:**
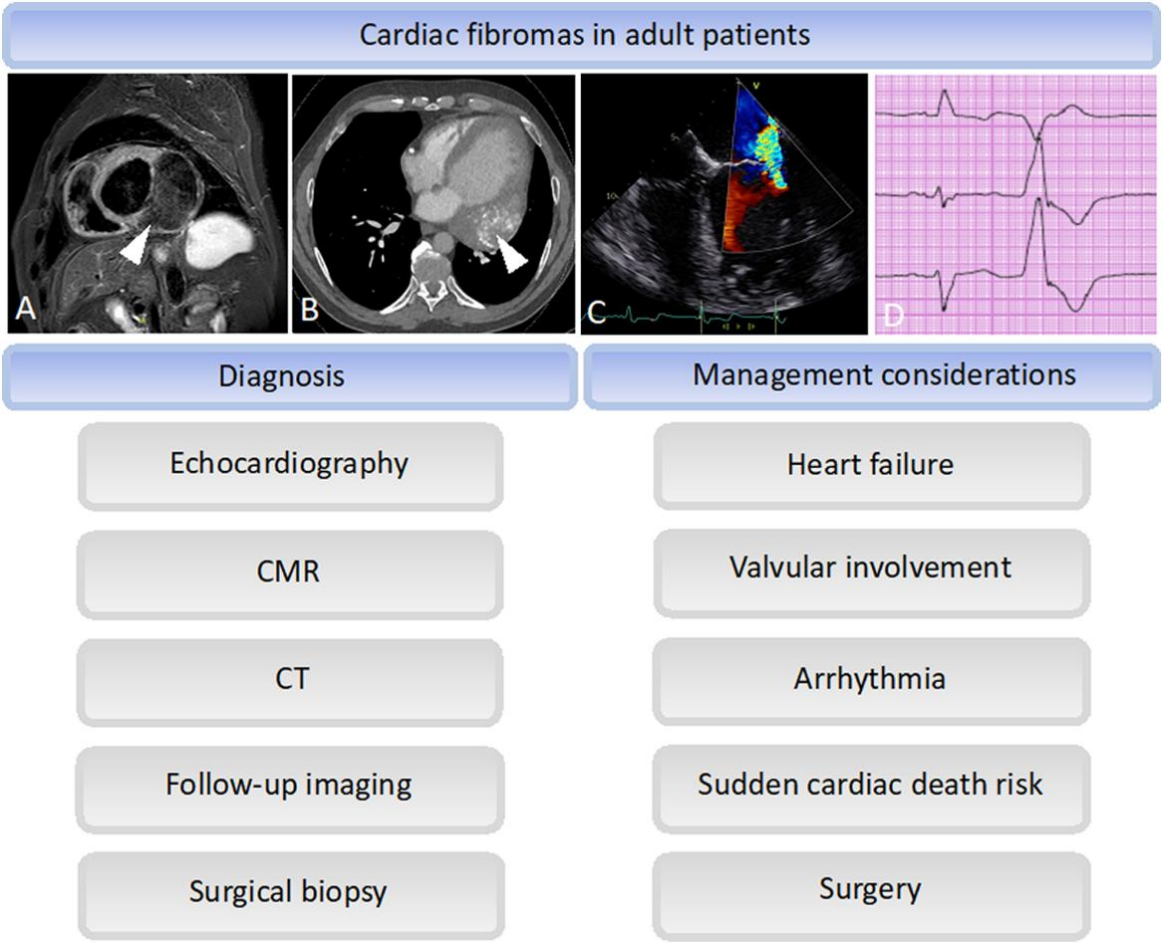
Diagnosis and management considerations of cardiac fibromas in adult patients. Fibromas were diagnosed by CMR in all cases (*A*, example from Case 1). CT supported the diagnosis in Cases 2 and 3 (*B*, example from Case 1). Fibroma led to valvular regurgitation in Case 1 with mitral regurgitation detected by transoesophageal echocardiography (*C*, colour Doppler regurgitation jet over the mitral valve). Premature ventricular contractions were present in Cases 1 and 2 (*D*, example from Case 1). CMR, cardiovascular magnetic resonance; CT, computed tomography.

### Patient 1

A 55-year-old male patient with dilated cardiomyopathy of unclear aetiology was referred to our tertiary care medical centre for evaluation. Family history included sudden death of the patient’s father after exercise at the age of 60 and sudden death of the mother at the age of 58.

Upon admission, the patient reported a limited exercise tolerance with dyspnoea [New York Heart Association (NYHA) functional class II]. Clinical examination was unremarkable except for a systolic murmur over the cardiac apex. Electrocardiogram (ECG) showed a sinus rhythm and a left bundle branch block with a QRS width of 150 ms. Coronary angiography revealed non-obstructive coronary artery disease.

**Figure 1:**
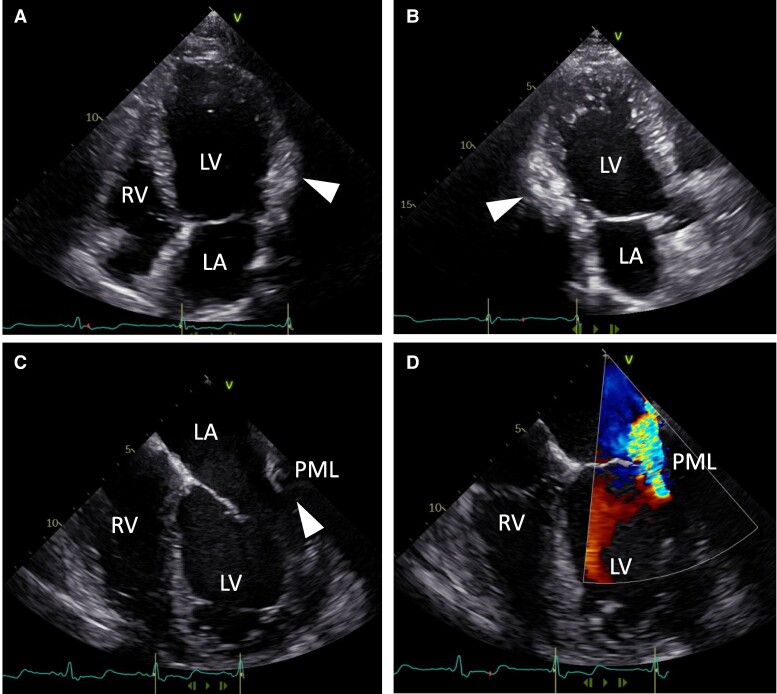
Transthoracic echocardiography showing only the margins of the left ventricular mass (arrowheads) in the apical four-chamber (*A*) and three-chamber (*B*) views. Transoesophageal echocardiography delineates the tethering (arrowhead, *C*) of the mass to the posterior mitral leaflet (PML) causing mitral regurgitation (Doppler jet, *D*). RV, right ventricle; LV, left ventricle; LA, left atrium.

Transthoracic echocardiography (TTE) revealed reduced left ventricular ejection fraction (LVEF) of 31%. Furthermore, margins of a hyperechogenic structure were seen in the basal segments of the left ventricular wall. Additionally, severe mitral regurgitation was detected. Complementary transoesophageal echocardiography revealed severe functional mitral valve regurgitation due to tethering of the mass to the posterior mitral leaflet (*Figure 1*).

**Figure 2:**
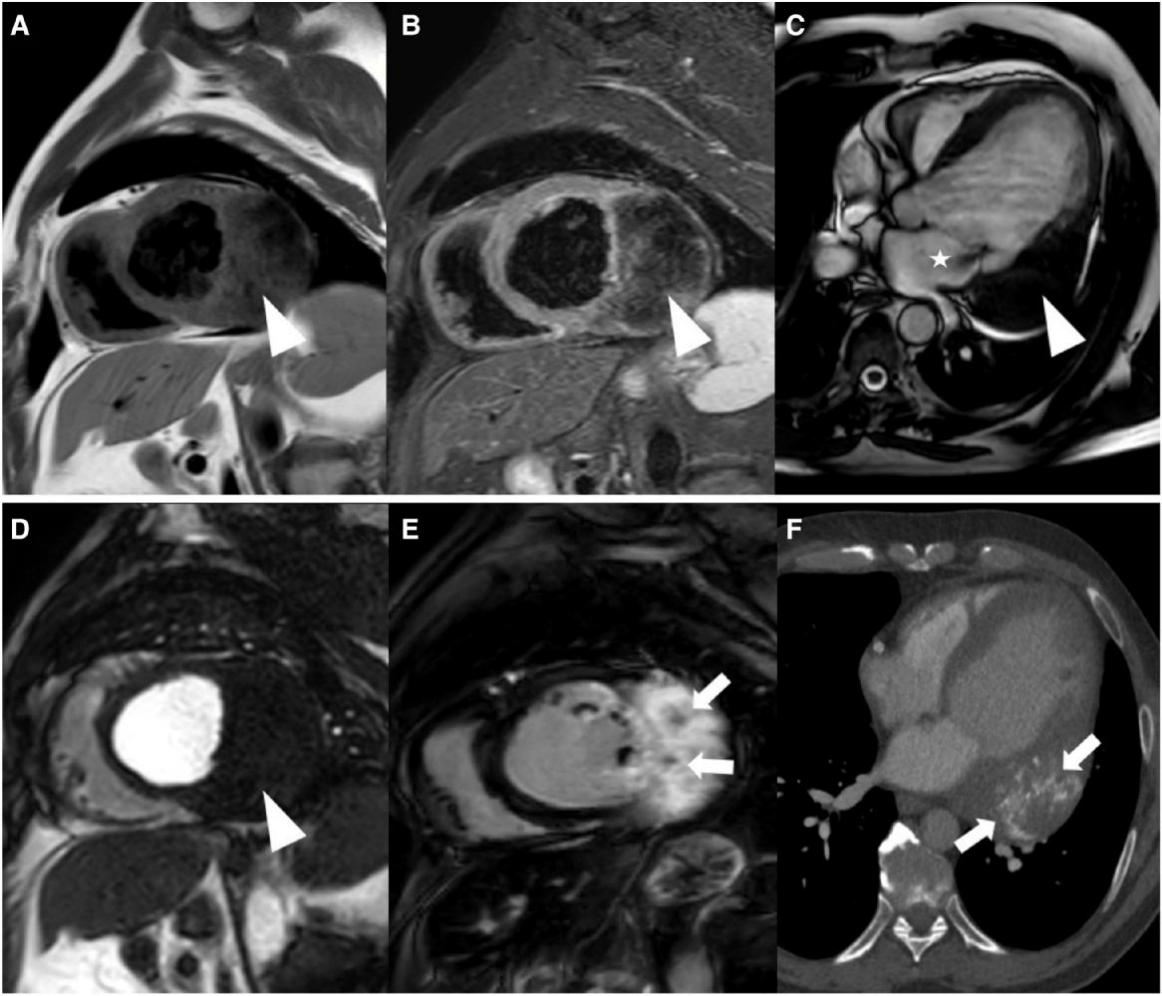
Cardiovascular magnetic resonance showing a semicircular cardiac mass of the left ventricular lateral wall with an iso- to hypointense signal in T1- (*A*, arrowhead) and hypointense signal in T2-weighted (*B*, arrowhead) sequences in short axis. Cine sequence in four-chamber view revealed an iso- to hypointense signal of the mass (*C*, arrowhead) and an attachment to the posterior mitral valve leaflet with associated insufficiency (*C*, asterisk). There was no hypervascularization of the mass in the first-pass-perfusion study in short axis (*D*, arrowhead). Late gadolinium enhancement (LGE) in short axis revealed a strong enhancement of the mass with spotty areas of calcification (*E*, arrows). CT revealed corresponding dystrophic calcifications (*F*, arrows). Overall, these features are consistent with a cardiac fibroma.

Guideline-directed heart failure medication (sacubitril/valsartan, bisoprolol, eplerenone, dapagliflozin) as well as a statin and aspirin was prescribed.^11^ Furthermore, the patient was referred for a cardiovascular magnetic resonance (CMR) study, which revealed a large intramural mass of 10.0 × 5.8 × 8.0 cm in the basal and midventricular left ventricular free wall adjacent to the mitral valve annulus with hypokinesia of the affected segments with a reduced LVEF of 26%. In complementary computed tomography (CT), the mass revealed dystrophic calcifications. Overall, these findings were consistent with a cardiac fibroma (*Figure 2*).

**Figure 3:**
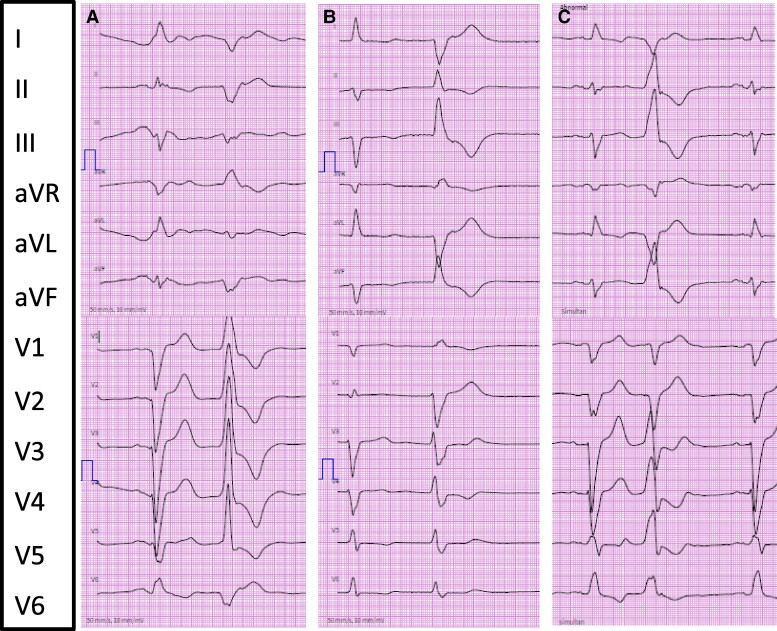
Twelve-lead ECG showing premature ventricular contractions. (*A*) In inferior left AV-region/apical morphology. Right-bundle branch block morphology with superior axis in II, III, and aVF. (*B*) In left-ventricular-outflow-tract morphology/LV-Summit. Right-bundle branch block morphology with inferior axis in II, III, and aVF. (*C*) In left-ventricular-outflow-tract morphology. Left-bundle branch block morphology with inferior axis in II, III, and aVF, Yoshida-index < 1.5. *A* and *B* represent the dominant morphologies.

The patient reported improvement of symptoms without any relevant dyspnoea upon exercise (NYHA I) after the start of heart failure medication. Based on interdisciplinary consensus, a surgical resection deemed not feasible. Left- and right heart catheterization revealed no haemodynamic significance of the mitral regurgitation at rest. Holter-ECG revealed polymorphic premature ventricular contractions (PVCs) with a burden of 17% and two dominant morphologies (*Figure 3*). Follow-up CMR after 6 months revealed no progression of the cardiac fibroma.

Due to the polymorphic PVCs, reduced LVEF, family history of sudden death, and the increased risk of ventricular arrhythmias and sudden cardiac death associated with the fibroma the decision was reached, together with the patient, to implant a primary preventive subcutaneous implantable cardiac defibrillator (S-ICD). After implantation, the patient was asymptomatic and is scheduled for regular follow-up examinations including TTE and Holter-ECG and the patient was advised against contact sports.

### Patient 2

**Figure 4:**
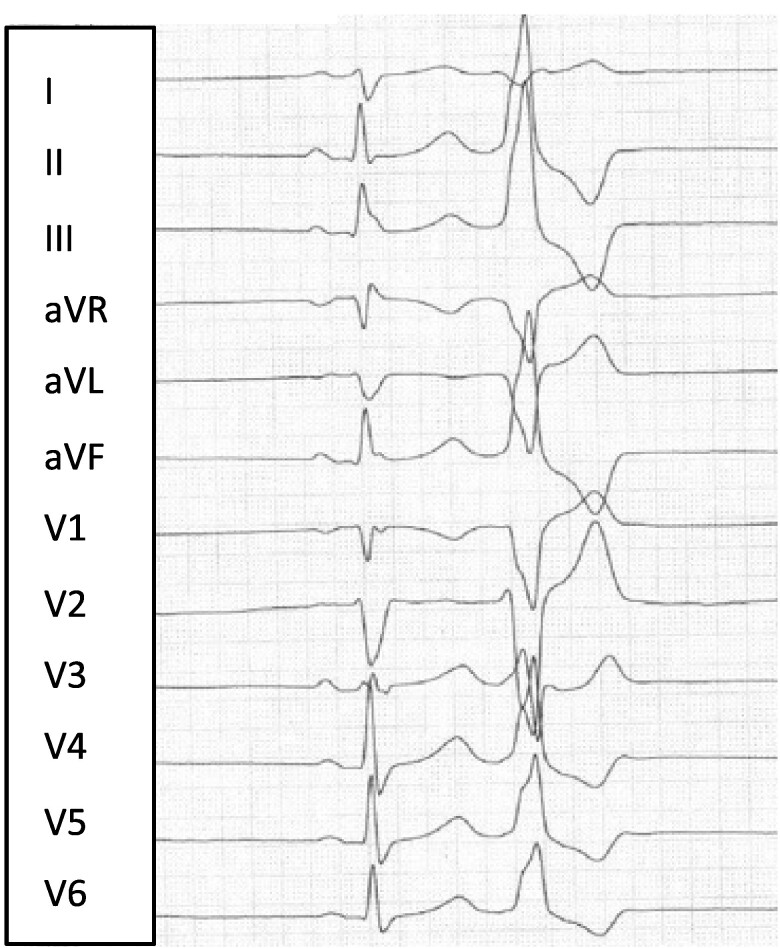
Twelve-lead ECG showing premature ventricular contractions in right ventricular outflow tract morphology. Left-bundle branch block morphology with inferior axis in II, III, and aVF.

A 39-year-old patient presented himself to a private practice due to an intermitted episode with palpitations and dizziness over several days. Electrocardiogram revealed a relevant PVC burden of 50% consistent with a right ventricular outflow tract morphology (*Figure 4*). Regarding the patient’s history, a right ventricular mass (2.7 × 1 cm) was first detected at the age of 18 years as an incidental finding and followed up regularly using TTE without showing progression. Left ventricular ejection fraction was always normal. A CMR study was not yet performed. The patient was referred to our tertiary care medical centre for evaluation of a PVC-ablation procedure. Holter-ECG at this time revealed only few PVCs with a burden of <0.1%.

**Figure 5:**
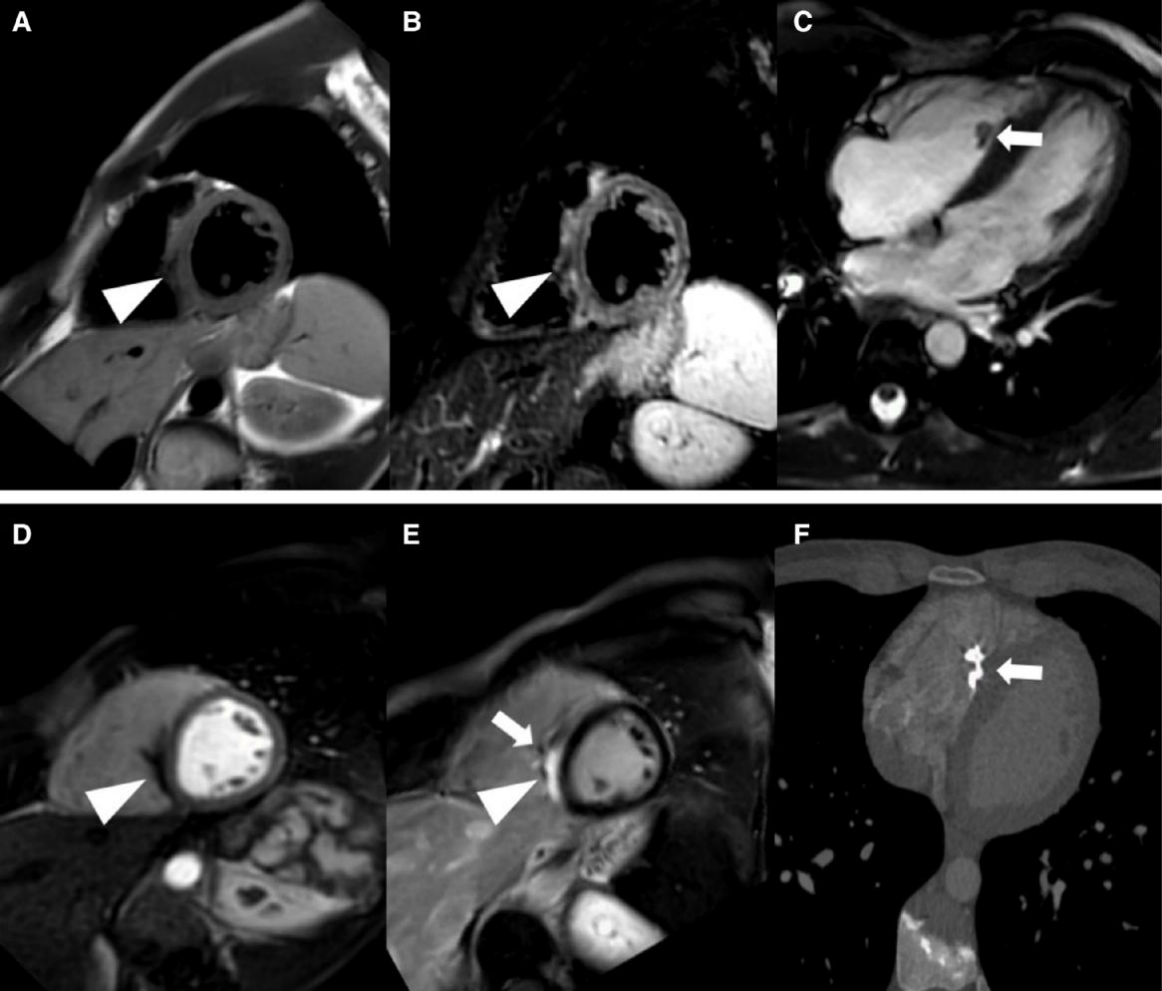
Cardiovascular magnetic resonance showing a broad-based cardiac mass of the right ventricular septal wall with an iso- to hypointense signal in T1- (*A*, arrowhead) and hypointense signal in T2-weighted (*B*, arrowhead) sequences in short axis. Cine sequence in four-chamber view revealed an iso- to hypointense signal (*C*, arrow). There was no hypervascularization of the mass first-pass-perfusion study in short axis (*D*, arrowhead). Late gadolinium enhancement revealed a homogeneous enhancement in short axis (*E*, arrowhead) with adjacent hypointense structures (*E*, arrow). Subsequent CT revealed corresponding dystrophic calcifications in this position (*F*, arrows). Overall, these features are consistent with a cardiac fibroma.

For further evaluation of the right ventricular mass, a CMR study was performed revealing a mass of 2.9 × 0.9 × 0.9 cm of the right ventricle attached to the basal and midventricular interventricular septum with strong late gadolinium enhancement (LGE). Adjacent to the mass, a linear structure with hypointense signal compared to the myocardium was detected. In suspicion of thrombus apposition, oral anticoagulation with apixaban was initiated. A complementary CT with a native scan revealed a calcified mass (*Figure 5*). Based on interdisciplinary consensus, the mass was classified as a cardiac fibroma.

Follow-up CMR after 6 months showed no change of the right ventricular mass and its hypointense adjacent structures, respectively, with the anticoagulation being terminated since thrombus apposition was deemed unlikely. Due to only an intermittent episode of symptomatic PVCs, decision was made against ablation. The patient is scheduled for regular follow-up with CMR.

### Patient 3

Patient 3 was an 18-year-old female born with dysmorphia syndrome. At birth, a left ventricular aneurysm was suspected on TTE (normal LVEF). Cardiovascular magnetic resonance at the age of 10 months revealed a partially inhomogeneous, diffusely contrast-enhancing mass of 4 cm at the cardiac apex. At that time, it was not possible to distinguish between a thrombosed aneurysm and a tumorous growth. Therefore, surgical biopsy was performed revealing the histological features of a cardiac fibroma. No obstruction of the left ventricular outflow tract and no cardiac symptoms such as dyspnoea or syncope were reported. Electrocardiogram showed T-wave inversions in the precordial leads V3–V6.

**Figure 6:**
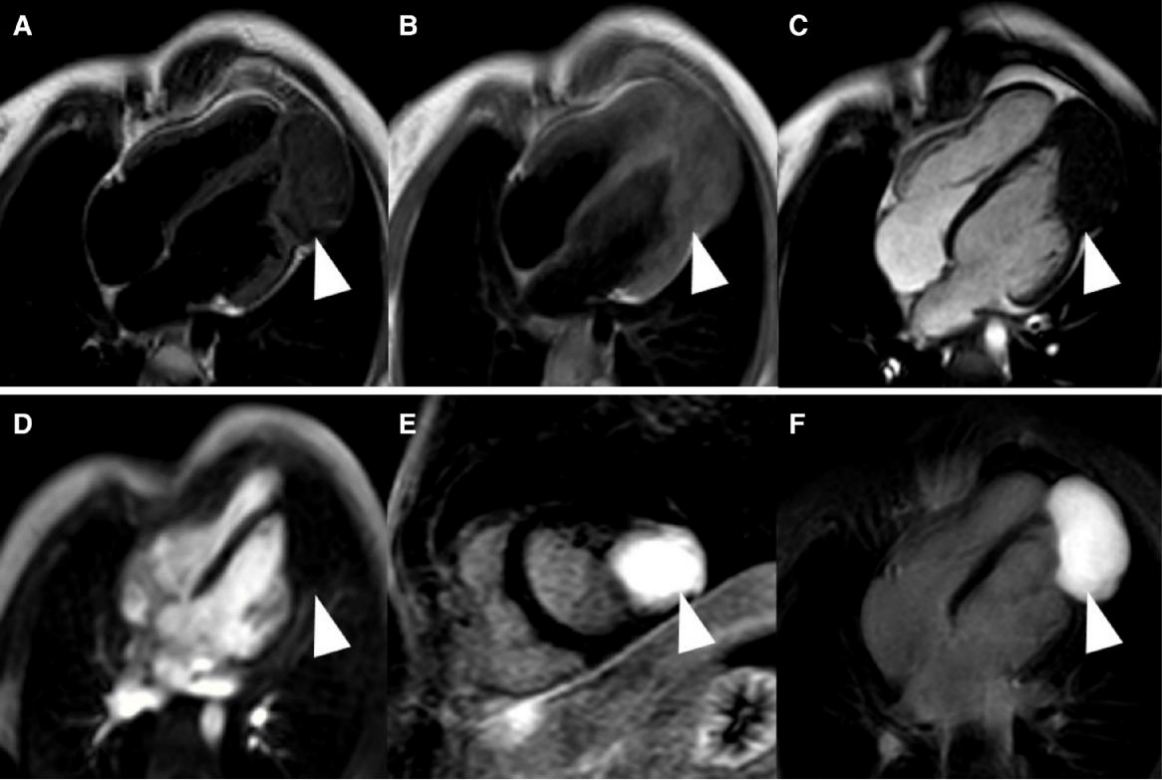
Cardiovascular magnetic resonance showing the fibroma of left ventricular apex with an iso- to hypointense signal in T1- (*A*, arrowhead) and iso- to hypointense signal in T2-weighted (*B*, arrowhead) sequences in four-chamber view. Cine sequences in four-chamber view revealed an iso- to hypointense signal (*C*, arrowhead). There was no hypervascularization of the mass in the first-pass-perfusion study in four-chamber view (*D*, arrowhead). Late gadolinium enhancement revealed a homogeneous enhancement in short axis (*E*, arrowhead) and four-chamber view (*F*, arrowhead) without calcifications.

At the age of 10 years, another CMR study was performed at our tertiary care medical centre for follow-up revealing the fibroma of the left ventricular apex with a size of 4.0 × 2.7 × 4.5 cm and homogenous late enhancement without hyperperfusion. The last follow-up MRI at the age of 14 showed slight progression of the fibroma to a size of 4.4 × 3.2 × 4.5 cm (*Figure 6*) with no clinical symptoms. Further follow-ups to the present day remained unchanged.

## Discussion

In this case series, we report three cases of cardiac fibromas in adult patients with distinct imaging features and arrhythmias. The cases are representative for the common locations of fibromas and various clinical phenotypes, ranging from asymptomatic to symptomatic presentations with severe impairment of cardiac function and rhythm disturbances.

The first case resembles the largest fibroma located in the basal and midventricular left ventricular wall with impairment of LVEF and mitral valve function. There are several reports indicating disturbance of valvular function by cardiac fibromas including causing mitral valve prolapse or regurgitation by directly infiltrating the leaflets.^9,12^ Invasive haemodynamic evaluation showed no significance of the mitral valve regurgitation at rest. Given the improvement of symptoms after the commencement of heart failure medication, surgical or interventional treatment was renounced. The reduced LVEF is most likely due to mechanical origin given the hypokinesia of the affected segments being infiltrated by the cardiac fibroma. It is unclear if studies on heart failure medication included cardiac fibromas as a cause of reduced LVEF. However, in the described case, the patient reported improvement of symptoms after heart failure medication. With a left bundle branch block of 150 ms and an LVEF of 31% in the present Case 1, guidelines would recommend an evaluation for cardiac resynchronization therapy (CRT) with defibrillator therapy.^11^ The left bundle branch block is most likely due to the fibroma and the effect of CRT in such cases is unknown with likely difficult placement of leads in the coronary sinus, therefore multidisciplinary consensus opted against CRT-placement.

Diagnostic workup on suspicion of a cardiac mass according to the 2022 ESC guidelines should include echocardiography, CMR, and CT.^13^ If there is a history of malignant cancer, additional positron emission tomography CT might be performed to rule out cardiac metastases that are more common than primary cardiac tumours.^13^ Furthermore, cardiac thrombi, as a mass itself or as thrombus apposition, as considered in Case 2, need to be excluded.^13^ Transthoracic echocardiography represents the initial imaging modality, which provides real-time images and helps to assess tumour size and location. On TTE, cardiac fibromas appear as well-defined, echogenic masses within the myocardium.^14^ Because of its superior soft tissue contrast, CMR is considered the non-invasive gold standard for evaluating cardiac masses. Relative to myocardium and due to their dense, fibrous nature, cardiac fibromas are usually hypointense on T2-weighted images and isointense on T1-weighted images. Given their avascularity, they usually show no hypervascularization in first-pass-perfusion but reveal homogeneous enhancement in LGE unless there are calcifications, which may be seen as patchy hypointense foci within the mass.^15^ The fibromas of the present case series yielded comparable signal intensities and contrast enhancement in CMR with Cases 1 and 2 showing calcifications. On CT, cardiac fibromas appear as non-enhancing, low density masses. Dystrophic calcifications are seen in ∼25% of cases and were present in Cases 1 and 2.^16^

Distinguishing cardiac fibromas from other cardiac masses such as rhabdomyomas, myxomas, thrombus, or lipoma is essential for accurate diagnosis and treatment planning, which may involve surgical biopsy, resection, or other interventions if findings are unclear as underlined in Case 3 in which the initial CMR study could not confirm cardiac fibroma.

Treatment of the described cases did not include surgical resection. Nevertheless, if fibromas become severely symptomatic or malignant ventricular arrhythmias are present, surgical resection is recommended if possible.^17^ In children, some authors recommend surgical excision even if asymptomatic due to the risk of malignant arrhythmias.^6^ After surgical resection, arrhythmias generally cease.^17^

Cardiac fibromas are described as being congenital thus even if detected in adults, they have probably been present since birth.^2^ There are theories that fibromas grow together with the heart and stop growing when the heart has reached its full size.^2^ Arrhythmia risk of adult patients without a history of syncope, symptomatic ventricular arrhythmias, or aborted cardiac arrest especially during exercise is unclear.

All our cases had no prior ventricular arrhythmias or history of syncope. No exercise restrictions were given in Cases 2 and 3. Patient 3 showed T-wave inversions. However, given mostly stable follow-up and with the fibroma being present since birth, conservative management with regular follow-up seemed warranted.

Patient 2 had palpitations and intermittent PVCs with a morphology associating them with the fibroma in the right ventricle. Cardiac fibromas are known to become apparent through PVCs^18^ that are associated with an increased risk of ventricular arrhythmias.^19^ In Case 2, the burden was low and the patient mostly asymptomatic with infrequent episodes, without the need for antiarrhythmic medication or an ablation. In Case 1, several factors prompted the implantation of an S-ICD. This included the large size of the fibroma, the reduced LVEF, the polymorphic PVCs with a high burden, and the family history of sudden death. In asymptomatic adult patients with cardiac fibromas, arrhythmia risk should be considered with individual assessment. Due to increasing utilization of CMR and CT, incidental findings of cardiac fibromas will likely increase potentially leading to more experience in management of cardiac fibromas in adult patients.

## Lead author biography


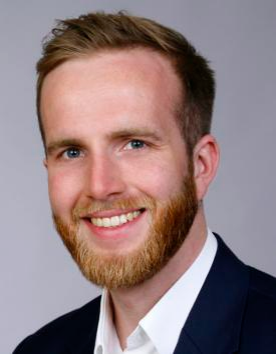


Karl Finke started his medical education at the Riga Stradins University in Riga, Latvia and later continued his education at the Friedrich-Schiller-University in Jena, Germany. After university, he spent a year working as a cardiology resident at the Örebro University Hospital (Sweden) and is currently completing his cardiology residency at the University Hospital Cologne (Germany). His research interests include cardiac critical care and cardiac imaging.

##  

**Consent:** Informed consent for publication of anonymized data was obtained from all patients who participated in this case series in line with COPE guidance.

**Funding:** No funding was received for this study.

## Data Availability

The data underlying this article cannot be shared publicly to protect the privacy of individuals that participated in this case series. The data will be shared on reasonable request to the corresponding author.
